# Selective Dorsal Rhizotomy for the Treatment of Spastic Hemiplegic Cerebral Palsy

**DOI:** 10.7759/cureus.9605

**Published:** 2020-08-07

**Authors:** TS Park, Susan Joh, Deanna M Walter, Nicole L Meyer, Matthew B Dobbs

**Affiliations:** 1 Neurosurgery, Washington University School of Medicine, St. Louis Children's Hospital, St. Louis, USA; 2 Pediatric Neurosurgery, Washington University School of Medicine, St. Louis Children's Hospital, St. Louis, USA; 3 Pediatric Orthopedic Surgery, Washington University School of Medicine, St. Louis Children's Hospital, St. Louis, USA

**Keywords:** selective dorsal rhizotomy, spasticity, hemiplegia, cerebral palsy, quality of life

## Abstract

Background

Selective dorsal rhizotomy (SDR) can remove spasticity in cerebral palsy (CP). Spastic hemiplegia is associated with spasticity in the upper and lower limbs on one side. Only a single report described the outcome of SDR specifically in patients with spastic hemiplegic CP. The effect of SDR on spastic hemiplegia requires further investigation.

Objectives

To analyze the outcomes of motor functions, the quality of life, and satisfaction of patients who received SDR for the treatment of spastic hemiplegia.

Methods

A total of 29 children and 1 adult who received SDR were surveyed. The survey questionnaire asked about demographic information, patient's perception of SDR, functional outcomes, SDR surgical outcomes, pain, braces/orthotics, and post-SDR treatment.

Results

Our study included 30 patients. The age at the time of surgery was 2 to 36 years. The follow-up period ranged from one to six years. Of all parents, 90% of parents reported that SDR benefited their children, and 93% stated that they would recommend the SDR procedure to other families of children with hemiplegic CP. Of all patients, 90% reported improved walking, 63% reported improved sitting, and 87% reported improved balance and posture. In daily life functioning after the SDR, 67% were more independent and confident. Moreover, 33% of patients were pain-free and 43% had reduced pain in their legs and back. In activities of daily living, 93% transferred independently from one position to another. A majority of the patients reported regular strengthening and stretching of the lower limb, and 50% of the patients played sports. A majority (73%) of patients underwent post-SDR orthopedic surgery for heel cord, hamstring, and adductor contractures. Five patients experienced numbness in the small part of the lower limb after SDR. None reported that the numbness affected their daily activities. One child required surgical repair of the cerebrospinal fluid leak.

Conclusions

In our 29 children and 1 adult with spastic hemiplegia, SDR improved motor function and daily life function. Nearly all parents of children and the one adult felt that SDR was beneficial and that they would recommend surgery to other children with spastic hemiplegia.

## Introduction

Currently, selective dorsal rhizotomy (SDR) is a recognized therapeutic intervention for spastic diplegia in many countries. SDR can eliminate spasticity and improve patient's mobility and the quality of life in diplegic patients. The beneficial effects of the operation extend into adulthood [1,2]. Amassed evidence in the literature and clinical experience with the procedure in the last four decades indicate the efficacy of SDR in the treatment of spastic diplegia [3]. By contrast, there was only a single publication on the outcome of SDR on children with spastic hemiplegia. Oki et al. described a reduction in spasticity and improvement in the quality of gait in 13 children followed for two years after SDR [4]. The present report describes our clinical experience with SDR on 30 total patients with spastic hemiplegia and surgical outcomes. The patients received unilateral SDR from 1988 to 2019 at the Center for Cerebral Palsy Spasticity at St. Louis Children’s Hospital, St. Louis, MO, USA.

## Materials and methods

The Institutional Review Board of Washington University School of Medicine approved this study. From a database with over 4,000 cerebral palsy (CP) patients at the Center for Cerebral Palsy Spasticity, we identified 63 patients who underwent unilateral SDR for hemiplegic CP between 1988 and 2018. We gathered their contact information from the database and medical records. We sent the study survey to the potential participants through email or through postal mail to those without current email addresses. The survey questions included demographic information, quality of life, perception of health, perception of the SDR procedure, motor and ambulatory functions, braces and orthotics, side effects of SDR, and post-SDR treatment. Questions about the adverse impact of SDR included bladder function and numbness and sensory change. Also, scoliosis and post-SDR orthopedic interventions were assessed. The SF-36 (36-Item Short Form) health survey was used to evaluate the perception of one's health [5]. Yes/no/not sure questions asked participants if they benefited from SDR and if they would recommend the procedure to other hemiplegic patients. They also had the option to add any comments about their perception of SDR.

We evaluated the quality of life of the patients. The items included receiving help with transferring positions, strengthening muscles and stretching various muscles (hamstrings, heel cords, adductors, affected arm), patients' independence since SDR, and engagement in recreational sports.

Patients were given the option to answer with improved/no change/worsened to questions about motor function, including sitting, balance when standing, and posture when standing. The ambulatory function was assessed by asking patients to indicate their level of walking before and after SDR.

## Results

Study cohort

By the time of the survey in 2019, 63 patients received a unilateral SDR for spastic hemiplegia. Of the 63 eligible patients, a total of 30 patients responded and consented to participation in the study, resulting in a 48% response rate. The ages at the time of surgery were 2.5 to 36.3 years. The follow-up period ranged from 1.0 to 6.8 years. The ages at the time of the survey were 4.1 to 39.4 years, with 7 children between 4 and 7 years of age, 22 children between 8 and 18 years of age, and 1 adult at 39 years of age.

Patient's perception of SDR

Of the 30 patients, 27 (90%) patients’ parents believed that their child benefited from SDR. Three parents were not sure, and the children of these parents had mild spasticity before the operation. The long-term benefit was the primary goal of SDR in the children. Moreover, 28 (93%) parents stated that they would recommend the SDR procedure to other families of children with hemiplegic CP (Table 1). All 30 patients reported that their health was good or better; a majority of the patients felt that their health was excellent.

**Table 1:** Patient Perception of SDR in 30 spastic hemiplegic patients SDR, selective dorsal rhizotomy

Did SDR benefit your child?	No. of patients (%)
Yes	27 (90%)
No	0
Not sure	3 (10%)
Would you recommend SDR to other families of children?	No. of patients (%)
Yes	28 (93%)
No	0
Not sure	2 (7%)

Improvement in motor function

Overall, motor function improved after SDR. No patient reported a worsened ability to sit, balance, or walk after the SDR procedure (Table 2). Of the 30 patients, 27 (90%) improved in their ability to walk; a majority of those patients are continuing to improve. Six of the seven patients who were able to walk in all environments could run after the SDR procedure.

**Table 2:** Motor function after SDR in 30 spastic hemiplegic patients SDR, selective dorsal rhizotomy

Outcomes of motor function	No. of patients (%)		
	Improved	No change	Worsened
Sitting	19 (63%)	11 (37%)	0
Balance	26 (87%)	4 (13%)	0
Posture	26 (87%)	3 (10%)	1 (3%)
Walking	27 (90%)	3 (10%)	0

Nineteen (63%) patients reported that their sitting has improved, and 26 (87%) reported improvement in balance and posture. One patient who could run before SDR reported worse posture, but sitting improved after the surgery.

Improvement in daily life function

When asked about the perception of changes in daily life functioning after the SDR, 20 (67%) were more independent and confident (Table 3). Ten (33%) patients were pain-free, and 13 (43%) had reduced pain in their legs and back. Ten (33%) patients performed better in school, and five (17%) improved in their ability to use the toilet.

**Table 3:** Perception of daily life function after SDR in 30 spastic hemiplegic patients SDR, selective dorsal rhizotomy

Improvement in daily life function	No. of patients (%)
Improved independence	20 (67%)
Improved confidence	20 (67%)
Pain-free in legs and back	10 (33%)
Reduced pain in legs and back	13 (43%)
Improved performance in school	10 (33%)
Improved ability to use a toilet	5 (17%)

Comments of Parents and Patients

“Less falls, improved balance and control of affected limb; She can keep up with her peers; Improved walking; Improved balance. Initially, improved speech intelligibility as well; She demonstrates a much more natural walking gait; She runs on her school's cross country team, takes ballet, tap/jazz, and rides a two-wheeled bike without training wheels. We do not believe she would be able to do any of these things without the surgery!; Better balance and stability, can jump and run; Stronger, progressing well in goals; Improved balance and flexibility; Less tightness in leg, legs are stronger; Improved gait and flexibility; I can participate in martial arts and do everything better; Her gait pattern improved, her balance improved, her leg discrepancy is decreasing, no more day braces, no more physical therapy; Muscles in right calf are developed and strong (they were small before surgery), gait has improved; he can lift toes somewhat and has feeling on that side (before he could not move toes or had much sensation; Balance is better, no brace is used; Improved flexibility; Spasticity in lower body is gone, muscles have been allowed to develop somewhat normally; He has a lot easier to walk and run; No spasticity in leg, not waking at night, improved balance and confidence, better foot placement, improved gait; There's not a lot of difference, but she can sit on a chair and cross her legs. She couldn't do that before; One thing we noticed is that she can now cross her legs when sitting down; better strength, better balance, better gait pattern; Child's gait has improved. Child can stand on whole foot rather than just the side of the foot. Child is able to keep up with the rest of the family. Better balance, increase in endurance, less tripping, no more toe walking, no more pain in the morning; Improved mobility; She can sit with legs crossed, her gait is better, she is no longer in pain, she has gained muscle on the right side of her body that she didn't have before; Improved posture, balance, strength; Improved balance, walking, right side functionality generally; Improved every ways; He walks better, his balance is bigger, he doesn't look handicapped at all; Mobility has improved and no longer in pain every day.”

Daily life activities

Patients were asked a series of questions about their activities of daily living after SDR. Twenty-eight (93%) patients did not need help with transfers from one position to another. As regular stretching and exercise of the affected muscles are crucial after SDR, a majority of the patients reported regularly strengthening muscles and stretching hamstrings and heel cords. Half of the patients were currently enjoying participating in recreational sports, including football, soccer, wrestling, swimming, and dancing (Table 4).

**Table 4:** Daily life activities after SDR in 30 spastic hemiplegic patients SDR, selective dorsal rhizotomy

Daily life activities	No. of patients (%)
Need assistance with position change	2 (7%)
Regularly strengthen muscles	26 (87%)
Regularly stretch hamstrings	22 (73%)
Regularly stretch heel cords	24 (80%)
Stretch adductors	14 (47%)
Stretch affected arm	15 (50%)
Play recreational sports	15 (50%)

Post-SDR orthopedic surgery, Botox injection, and medication 

Scoliosis and other back issues were observed in 10% of patients, and no patients received treatment for these issues. None of the patients received hip surgery as well. One patient received a bone surgery, but it was not a derotational osteotomy. A majority (73%) of the patients received a tendon lengthening surgery. The procedure is required for patients with contractures in their joints along with the SDR, as SDR only relieves spasticity. Fifteen of those patients received the surgery from Dr. Matthew Dobbs. Nine patients received the surgery on their hamstrings, 18 on Achilles tendon, three on adductors, and none on calf muscles.

Some patients received other non-surgical treatments. No patient had a Baclofen pump before or after the SDR procedure. Two (7%) patients received Botox injections to treat spasticity in the leg. No patients took medications for their spasticity (Table 5).

**Table 5:** Post-SDR orthopedic surgery, Botox injection, and medication in 30 spastic hemiplegic patients *Each patient may have received more than one kind of surgery. SDR, selective dorsal rhizotomy

Post-SDR orthopedic surgery*	No. of patients (%)
Bone surgery	1 (3%)
Tendon-lengthening surgery	22 (73%)
Hamstrings	9 (30%)
Achilles tendon	18 (60%)
Adductors	3 (10%)
Calf muscles	0
Botox injection for spasticity in the leg	2 (7%)
Medications for spasticity	0

Postoperative orthotics

Sixty percent of children were using braces at the time of the survey. Thirteen patients used ankle-foot orthosis (AFOs), seven patients used supra-malleolar orthotics (SMOs), one patient used UCBs. Six patients went from using braces to either not using them at all or only using them at night.

Side effects of SDR

Five (17%) patients experienced numbness in the small area of the lower limb after SDR. The numbness was in the L2, L3, L5, or S1 dermatomes. No patient felt a complete loss of sensation in the lower limb. None reported that the numbness affected their daily activities. One child resumed active independent walking in a week after surgery. She engaged in excessive physical activities and developed a sudden cerebrospinal fluid leak. She required surgical repair of the leak. No patients experienced urinary incontinence or scoliosis as a result of the SDR.

Case report

This 11-year-old girl with right-sided spastic hemiplegic CP underwent SDR at seven years of age. On the pre-SDR examination, she showed equinovarus foot deformity, hamstring, and heel cord contractures. She received an Achilles tendon lengthening procedure two weeks after SDR. She improved ambulation (Video 1). She no longer uses braces and discontinued physical therapy.

**Video 1:** Pre- and post-operative comparison of SDR Permission was given by the parents to display patient identifying information in the video. SDR, selective dorsal rhizotomy

## Discussion

A total of seventy-four patients received treatment with SDR for spastic hemiplegia, which constitutes only 2% of the 4,336 patients treated at our center between 1987 and 2019. The small number of hemiplegic patients is due to the lower prevalence of spastic hemiplegia and our practice bias in the early years. Until 15 years ago, we did not recommend SDR for hemiplegic patients. The reason was that all spastic hemiplegic patients walk independently, and we were unsure if SDR could significantly benefit the already functioning patient group. As we gained more experience with the harmful effects of CP spasticity and the outcomes of SDR in spastic diplegic patients [1,2], we changed our SDR practice. We began to offer SDR to patients with spastic hemiplegia.

Concerning the rationale for SDR on hemiplegic patients, it is essential to note that there is currently no proven benefit of spasticity in CP. By contrast, the adverse effects of CP spasticity are well known. Ideally, spasticity must be removed to prevent the life-long harmful effects. Spasticity inhibits voluntary movement, muscle stretch, and longitudinal muscle growth. In an experimental study, longitudinal muscle growth was reduced by 45% in spastic mice compared with control animals [6]. The longitudinal muscle growth occurs during development as the new sarcomeres develop in the muscle fibers [7]. The increase in the sarcomeres is not under neural control but induced mainly by the amount of tension on the muscle, i.e., repeated muscle stretch [8]. Thus, spasticity results in a decline of new sarcomeres in the muscle cells and loss of elasticity. The muscle contracture, i.e., increased resistance of the muscle to passive stretch in the absence of muscle contraction, follows [9]. The muscle contractures typically worsen as a child grows and produce various bone and joint deformities in the extremities [10]. Also, spasticity damages muscle in children and adults [11,12]. Muscle injury leads to "early aging," which manifests as muscle weakness, loss of endurance, increasing deformities, and body aches. In our experience, early aging often begins around 10 years of age in childhood and at any age in adulthood. Most significantly, the eventual outcome of the early aging process is the loss of the ability to walk in late adulthood [13].

We have found that nearly all hemiplegic patients walk independently in all or protected environments. However, if left untreated, these patients develop a multitude of disabilities due to spasticity and impaired motor function. The disabilities include inhibited walking and transition movements, low endurance and fatigue, impaired balance and frequent falls, pain, heel cord and hamstring contractures associated with toe walking and crouch knees, leg length discrepancy, hip subluxation, pelvic tilt, and limited ability to stretch and exercise. Many patients receive Botox injection and baclofen. They also need long ankle-foot devices (AFOs). Unless spasticity is removed, their functions decline as they age. Thus, spasticity must be removed. Fortunately, SDR can remove spasticity permanently. At our center, after SDR, the orthopedic deformities are treated with minimally invasive orthopedic surgery. After the combined treatments, the vast majority of patients improve motor functions and the quality of life. They can do strengthening exercise and use shoe inserts instead of an AFO. They do not need Botox injection and anti-spasticity medication.

All patients in this series underwent unilateral SDR on the side of involvement. Around 65% of dorsal rootlets on one side at each level of L1-S2 were divided as described earlier [14]. Many patients came to us with a diagnosis of spastic hemiplegia. However, we found them to harbor spasticity, and patients underwent bilateral SDR under the new diagnosis of spastic triplegia. All patients walked independently before surgery. After the unilateral SDR, postoperative recovery was always rapid to resume independent walking in less than 10 days. Some patients showed improvements in the upper limb movements except the finger movements. In young children, improvements from the SDR continue to about 10 years of age. In adolescents and adults, improvement time after the surgery is about two years. After the improvement period, patient outcome will mostly depend on continuing exercise and stretch.

## Conclusions

In our 29 children and 1 adult with spastic hemiplegia, SDR improved motor function and daily life function. Nearly all parents of children and one adult felt that SDR was beneficial and that they would recommend surgery to other children with spastic hemiplegia.
